# HOXC8 promotes breast tumorigenesis by transcriptionally facilitating cadherin-11 expression

**DOI:** 10.18632/oncotarget.1841

**Published:** 2014-03-22

**Authors:** Yong Li, Fengmei Chao, Bei Huang, Dahai Liu, Jaejik Kim, Shuang Huang

**Affiliations:** ^1^ Center for Stem Cell and Translational Medicine, School of Life Sciences, Anhui University, Hefei, Anhui, China; ^2^ Department of Biostatistics, Georgia Regents University, Augusta, GA, USA; ^3^ Department of Biochemistry and Molecular Biology, Medical College of Georgia, Georgia Regents University, Augusta, GA, USA; ^4^ E-institute of Shanghai Municipal Education Committee, Shanghai University of Traditional Chinese Medicine, Shanghai, China

**Keywords:** HOXC8, CDH11, breast cancer, transcription, metastasis

## Abstract

Cell-cell adhesion molecule cadherin-11(CDH11) is preferentially expressed in basal-like breast cancer cells and facilitates breast cancer cell migration by promoting small GTPase Rac activity. However, how the expression of CDH11 is regulated in breast cancer cells is not understood. Here, we show that CDH11 is transcriptionally controlled by homeobox C8 (HOXC8) in human breast cancer cells. HOXC8 serves as a CDH11-specific transcription factor and binds to the site of nucleotides −196 to −191 in the CDH11 promoter. Depletion of HOXC8 leads to the decrease in anchorage-independent cell growth, cell migration/invasion and spontaneous metastasis of breast cancer cells; however, suppressed tumorigenic events were fully rescued by ectopic CDH11 expression in HOXC8-knockdown cells. These results indicate that HOXC8 impacts breast tumorigenesis through CDH11. The analysis of publically available human breast tumor microarray gene expression database demonstrates a strong positive linear association between HOXC8 and CDH11 expression (*ρ* = 0.801, p < 0.001). Survival analysis (Kaplan-Meier method, log-rank test) shows that both high HOXC8 and CDH11 expression correlate with poor recurrence-free survival rate of patients. Together, our study suggests that HOXC8 promotes breast tumorigenesis by maintaining high level of CDH11 expression in breast cancer cells.

## INTRODUCTION

Homeobox C8 (HOXC8) is one of the 39-member HOX family proteins [1]. HOXC8-null mice exhibit neuromuscular defects in forelimb and skeletal defects in the ribs and vertebrae of the thorax [2] while overexpression of a HOXC8 transgene causes cartilage defects with an accumulation of proliferating chondrocytes and reduced maturation in skeletal elements [3]. These observations indicate that HOXC8 plays an essential role for neuromuscular and skeletal development [4]. Recent studies have also linked HOXC8 into the tumorigenesis of various cancer types. For example, HOXC8 expression is selectively turned on in human cervix cancer cells [5] and is associated with the loss of tumor differentiation in human prostate cancer cells [6]. We showed that HOXC8 level is elevated in invasive/metastatic breast tumor cell lines and its presence is required for breast cancer cell migration and metastasis [7]. However, the mechanism underlying HOXC8 regulation of tumorigenesis is little understood. In murine embryonic fibroblasts, HOXC8 was reported to influence the expression of a set of genes important for cell adhesion, migration and apoptosis [8-9]. It is of our interest to investigate whether one or more of these genes are functionally critical for tumorigenic events elicited by HOXC8.

Cadherin 11 (CDH11) belongs to the cadherin superfamily that comprises at least 6 subfamilies [10]. CDH11, also known as OB-cadherin, is originally identified in mouse osteoblasts and functions to mediate homophilic cell-cell adhesion in a calcium dependent manner [11]. During early embryogenesis, CDH11 is expressed predominantly in mesenchymal tissues but not in epithelia tissues [12-13]. The involvement of CDH11 in tumorigenesis was initially indicated by the observation that CDH11 was overexpressed in various cancer types including breast, prostate, osteosarcoma and colon cancers [6, 14-17]. Because CDH11 is often only detected in aggressive cancer cell lines and tissues [14, 18-19] and ectopically expressing CDH11 can effectively enhance cancer cell migration and metastasis [18, 20-21], CDH11 has been regarded as a metastasis promoter. To understand how CDH11 is involved in breast cancer cell migration, we previously showed that CDH11 was able to promote small GTPase Rac activity by facilitating the plasma translocation of Rac-specific GEF Trio, an event essential for Rac activation and cell migration in breast cancer cells [22]. Given the importance of CDH11 in breast tumorigenesis, there is little knowledge on how the expression of CDH11 is controlled in cancer cells.

The initial objective of this study is to understand how CDH11 expression is regulated in breast cancer cells. Here, we show that enforcing HOXC8 expression significantly upregulates CDH11 expression in breast cancer cells. We further show that HOXC8 promotes CDH11 expression by serving as a CDH11 transcription factor. HOXC8 binds to the site of nucleotides −196~-191 in the CDH11 promoter and site-directed mutagenesis at this site completely abolishes the ability of HOXC8 to activate CDH11 promoter. To functionally link HOXC8 to CDH11 in breast tumorigenesis, we show that the depletion of HOXC8 leads to the significant reduction in anchorage-independent cell growth, cell migration/invasion and spontaneous metastasis of breast cancer MDA-MB-231 and Hs578T cells. However, suppressed tumorigenic events are fully rescued by ectopically expressing CDH11 in HOXC8-knockdown cells. These results suggest that HOXC8 facilitates breast tumorigenesis by promoting HOXC8 expression. Analysis of breast tumor sample microarray database reveals that the expression HOXC8 and CDH11 are in a strong positive linear association. Kaplan-Meier analysis further shows that both high HOXC8 and CDH11 expression correlate with poor recurrence-free survival of breast cancer patients. This study indicates that the HOXC8-CDH11 axis plays a critical role in breast tumorigenesis.

## RESULTS

### HOXC8 promotes CDH11 expression by enhancing CDH11 transcription

**Figure 1:**
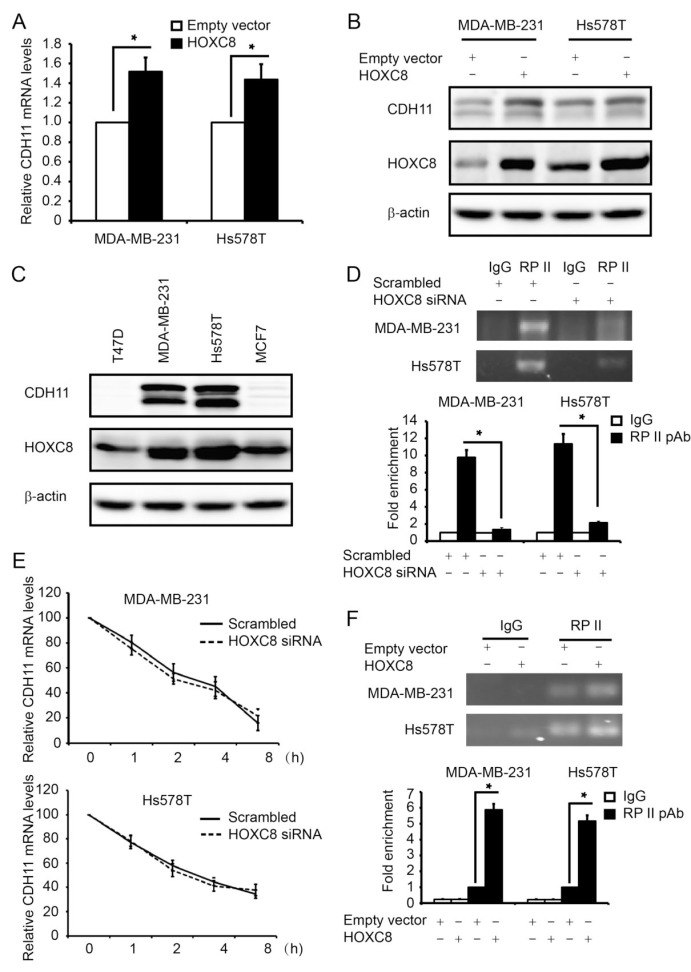
HOXC8 enhances CDH11 transcription in breast cancer cell lines (A) Total RNA was isolated from MDA-MB-231 or Hs578T cells lentivirally transduced with empty or HOXC8 expression vector, and then subjected to qRT-PCR to measure the level of CDH11 mRNA. GAPDH mRNA was used as an internal control for standardization. Columns, means; bars, SEM; *n* = 3. *, *P* < 0.05. (B) MDA-MB-231 or Hs578T cells lentivirally transduced with empty or HOXC8 expression vector were lysed and cell lysates were subjected to Western blot to detect HOXC8, CDH11, and β-actin with the respective antibodies. (C) Overnight cultured MDA-MB-231, Hs578T, MCF7 and T47D were lysed and cell lysates subjected to Western blot to detect HOXC8, CDH11 and β-actin with the respective antibodies. (D) MDA-MB-231 and Hs578T cells were transfected with scrambled or HOXC8 siRNA for 4 days and then subjected to ChIP with control IgG or RP II polyclonal antibody. The immunoprecipitated chromatin DNA was analyzed by PCR (upper panel) or qRT-PCR (lower panel) with primers amplifying region near the transcription start site (TSS) of the CDH11 promoter. Columns, means; bars, SEM; *n* = 3. *, *P* < 0.05. (E) MDA-MB-231 or Hs578T cells were transfected with scrambled or HOXC8 siRNA for 4 days followed by adding 2μg/ml actinomycin to the culture. Total RNA was isolated at varying times and then subjected to qRT-PCR to measure the level of CDH11 mRNA. GAPDH mRNA was used as an internal control. The level of CDH11 mRNA without actinomycin treatment was considered as 100%. Values are means ± SEM; *n* = 3. (F) MDA-MB-231 or Hs578T cells lentivirally transduced with empty or HOXC8 expression vector were subjected to ChIP with either control IgG or RP II polyclonal antibody. The immunoprecipitated chromatin DNA was analyzed by PCR (upper panel) or qRT-PCR (lower panel). Columns, means; bars, SEM; *n* = 3. *, *P* < 0.05.

We previously reported that knockdown of HOXC8 led to the reduction in CDH11 expression in invasive breast cancer cells [22]. To further define the role of HOXC8 in CDH11 expression, we ectopically expressed HOXC8 in breast cancer MDA-MB-231 and Hs578T cell lines. QRT-PCR showed that forced HOXC8 expression increased the amount of CDH11 mRNA in both cell lines (Fig.1A). Western blot analysis similarly showed that the level of CDH11 protein was elevated in cells with ectopic HOXC8 expression comparing to the empty vector-transduced control cells (Fig.1B). CDH11 level was apparently correlated with HOXC8 expression because breast cancer cell lines with CDH11 expression all displayed relatively higher HOXC8 level compared with those with little or no detectable CDH11 (Fig.1C). To elucidate molecular mechanism mediating HOXC8-regulated CDH11 expression, we initially analyzed the effect of HOXC8-knockdown on CDH11 transcription and mRNA stability in MDA-MB-231 and Hs578T cells. ChIP with RNA polymerase II (RP-II) pAb showed that knockdown of HOXC8 decreased the occupancy of RP-II in the CDH11 promoter by more than 80% (Figure 1D). In contrast, actinomycin-chasing experiment showed that silencing HOXC8 displayed no significant effect on stability of CDH11 mRNA (Fig.1E). In parallel, we also assessed the effect of forced HOXC8 expression on CDH11 transcription in MDA-MB-231 and Hs578T cells. ChIP showed that the occupancy of RP-II in the CDH11 promoter raised 4-6 folds in cells with forced HOXC8 expression over the control cells (Fig.1F). These results suggest that HOXC8 regulates CDH11 expression transcriptionally in breast cancer cells.

### HOXC8 activates CDH11 promoter in breast cancer cells

**Figure 2:**
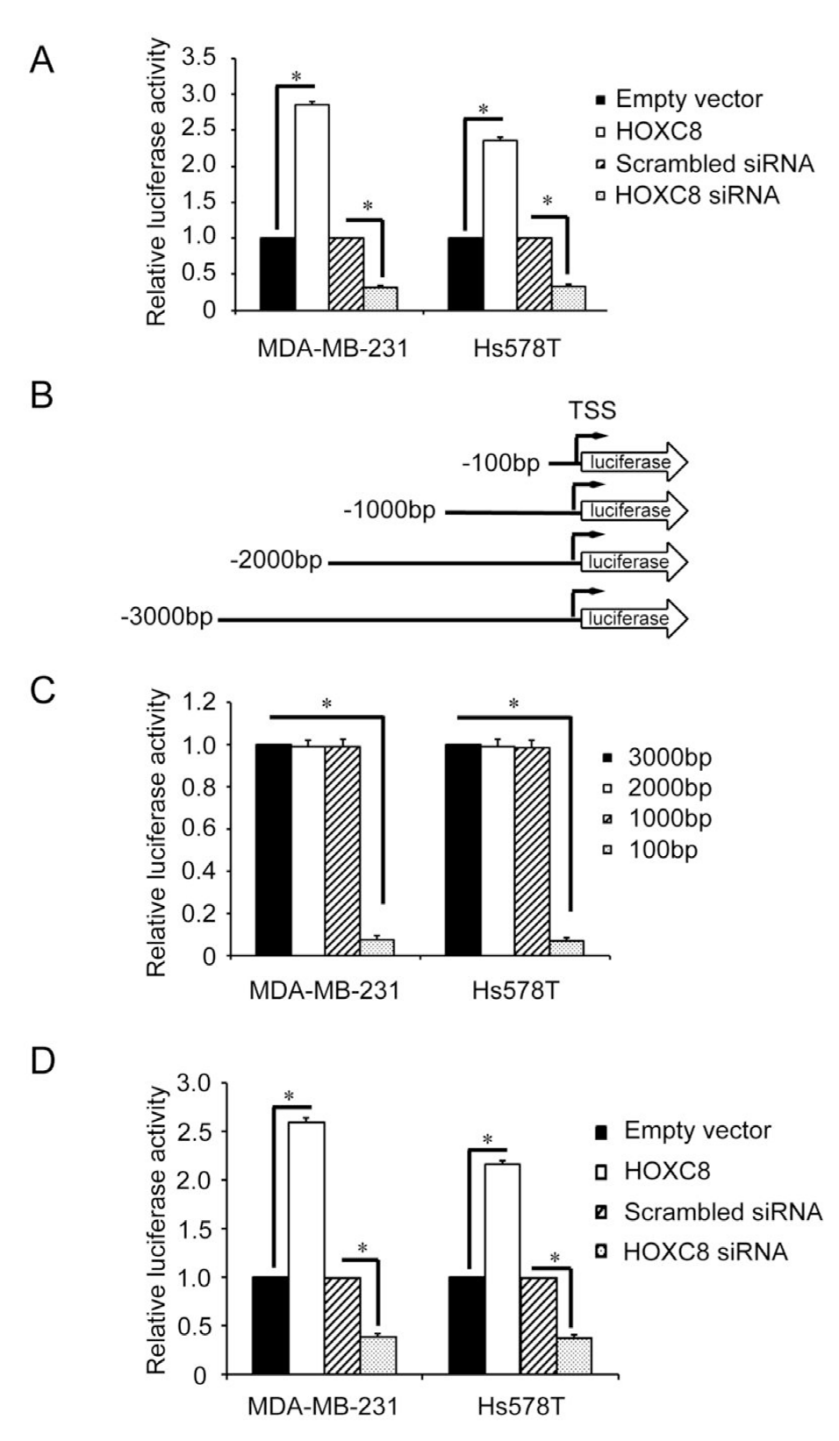
Deletion analysis of the CDH11 promoter (A) The 3,000-nucleotide CDH11 promoter luciferase reporter plasmid was transfected into MDA-MB-231 or Hs578T cells that were either transfected with HOXC8 siRNA or HOXC8 expression vectors. After 24 hrs, cells were lysed and lysates were analyzed for luciferase activities. pTK-Renilla luciferase plasmid was included in transfection for standardization. Columns, means; bars, SEM; n = 3. *, *P* < 0.05. (B) A series of 5'-deletion of CDH11 promoter in the pGL3-Basic vector were generated using PCR. TSS: transcription start site. (C) The different lengths of CDH11 promoter reporter plasmids were transfected into MDA-MB-231 or Hs578T cells for 24 hrs and then analyzed for luciferase activities. pTK-Renilla luciferase plasmid was included in transfection for standardization. Columns, means; bars, SEM; n = 3. *, *P* < 0.05. (D) The 1,000-nucleotide CDH11 promoter reporter plasmid was transfected into MDA-MB-231 or Hs578T cells that were previously transfected with HOXC8 expression vector or HOXC8 siRNA. After 24 hrs, cells were lysed and lysates were analyzed for luciferase activity. pTK-Renilla luciferase plasmid was included in transfection for standardization. Columns, means; bars, SEM; n = 3. *, *P* < 0.05.

The nature of HOXC8 as a transcription factor prompted us to hypothesize that HOXC8 serves as a CDH11 transcriptional factor. To test this hypothesis, we generated a CDH11 promoter reporter gene plasmid by cloning the 3,000-nucleotide region upstream of CDH11 transcription start site (TSS) into firefly luciferase gene-containing pGL2 vector. Analysis with this plasmid showed that knockdown of HOXC8 decreased luciferase activity while forcing HOXC8 expression enhanced luciferase activity in both MDA-MB-231 and Hs578T cells (Fig.2A). To identify the region in CDH11 promoter important for CDH11 transcription, we generated a series of CDH11 promoter deletion constructs (Fig.2B). Luciferase activity analysis showed that region of nucleotides −1,000~+1 displayed as strong activity as the 3,000-nucleotide region of the CDH11 promoter while region of nucleotides −100~+1 had less than 10% of the activity remaining (Fig.2C). These results indicate that the region of nucleotides −1,000~−100 contains *cis*-element critical for CDH11 transcription. In subsequent experiment, we examined how modulating HOXC8 level affected the activity of CDH11 promoter with this 1,000 nucleotide CDH promoter plasmid. Enforcing HOXC8 expression enhanced luciferase activities while silencing HOXC8 lessened luciferase activities (Fig.2D). These results suggesting that the region of nucleotides −1,000 to −100 in the CDH11 promoter is responsible for HOXC8 regulation of CDH11 transcription in breast cancer cells.

### HOXC8 binds directly to the CDH11 promoter

**Figure 3:**
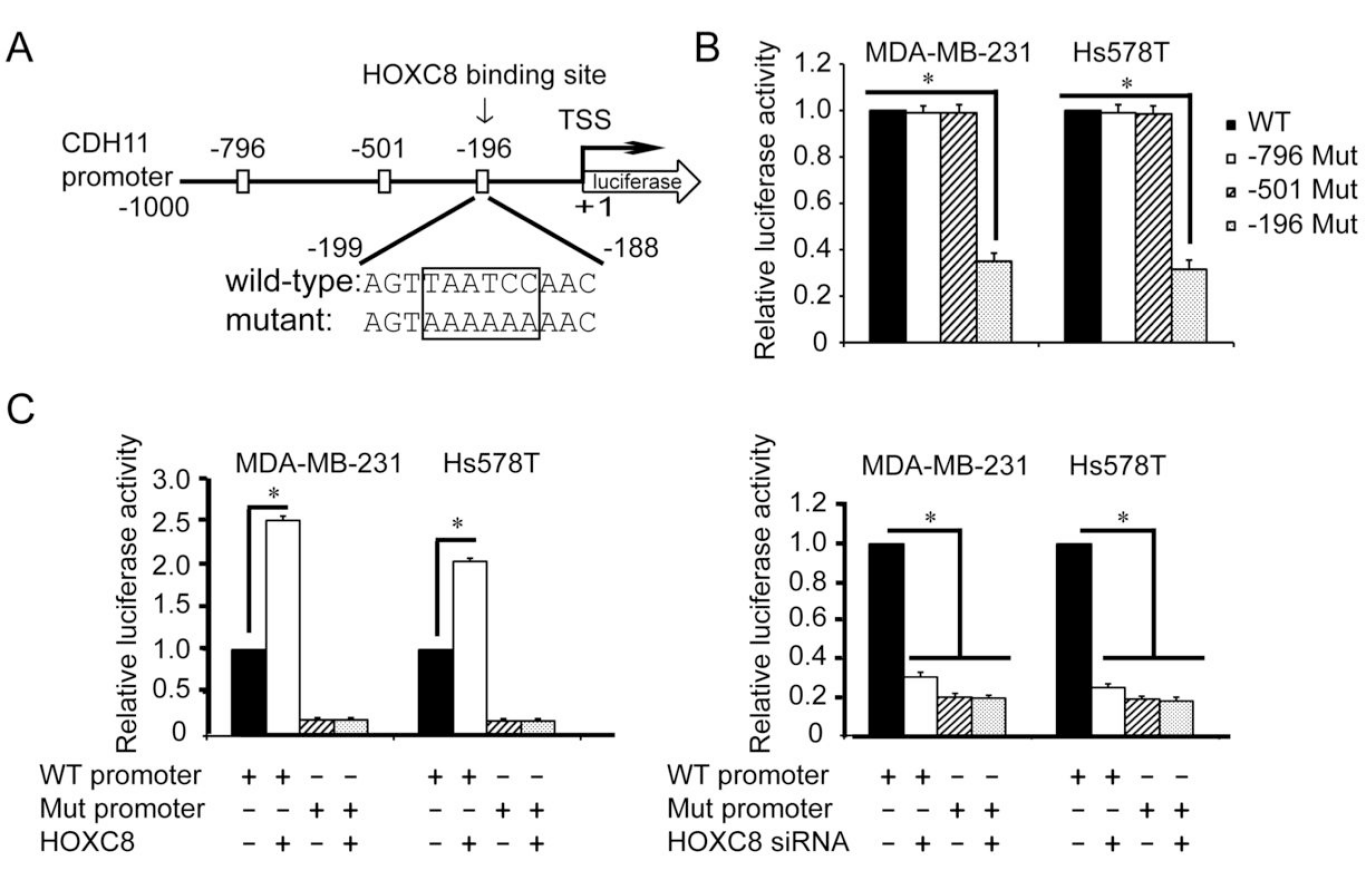
Region containing nucleotides −196~−191 is essential for HOXC8-induced CDH11 promoter activation (A) The positions of three putative HOX protein binding sites in CDH11 promoter region. TSS, transcription start site. (B) MDA-MB-231 or Hs578T cells were transfected with wild-type or various mutant CDH11 promoter reporter plasmid for 24 hrs followed by the analysis of luciferase activity. pTK-Renilla luciferase plasmid was included in transfection for standardization. Columns, means; bars, SEM; n = 3. *, *P* < 0.05. (C) The reporter plasmid containing 1,000-nucleotide wild-type CDH11 promoter or CDH11 promoter containing mutation at nucleotides −196~−191 was transfected into MDA-MB-231 or Hs578T cells that were previously transfected with HOXC8 expression vector (left panel) or HOXC8 siRNA (right panel). After 24 hrs, cells were lysed and lysates were analyzed for luciferase activities. pTK-Renilla luciferase plasmid was included in transfection for standardization. Columns, means; bars, SEM; n = 3. *, *P* < 0.05.

Based on the known HOX protein-binding consensus sequences TAATNN [1, 23], we identified 3 such sequences at nucleotides −796~−791, −501~−496 and −196~−191 in nucleotides −1,000~+1 of the CDH11 promoter (Fig. 3A). Mutagenesis at these sites followed by luciferase activity assay showed that only the mutagenesis at nucleotides −196~-191 diminished CDH11 promoter activity (Fig.3B). Moreover, CDH11 promoter containing mutation at nucleotides −196~−191, but not mutations at other two sites, was not responsive to either forced HOXC8 expression or HOXC8 knockdown (Fig.3C). These results suggest that HOXC8-regulated CDH11 transcription requires the sequence at the nucleotides −196~−191 in the CDH11 promoter.

**Figure 4:**
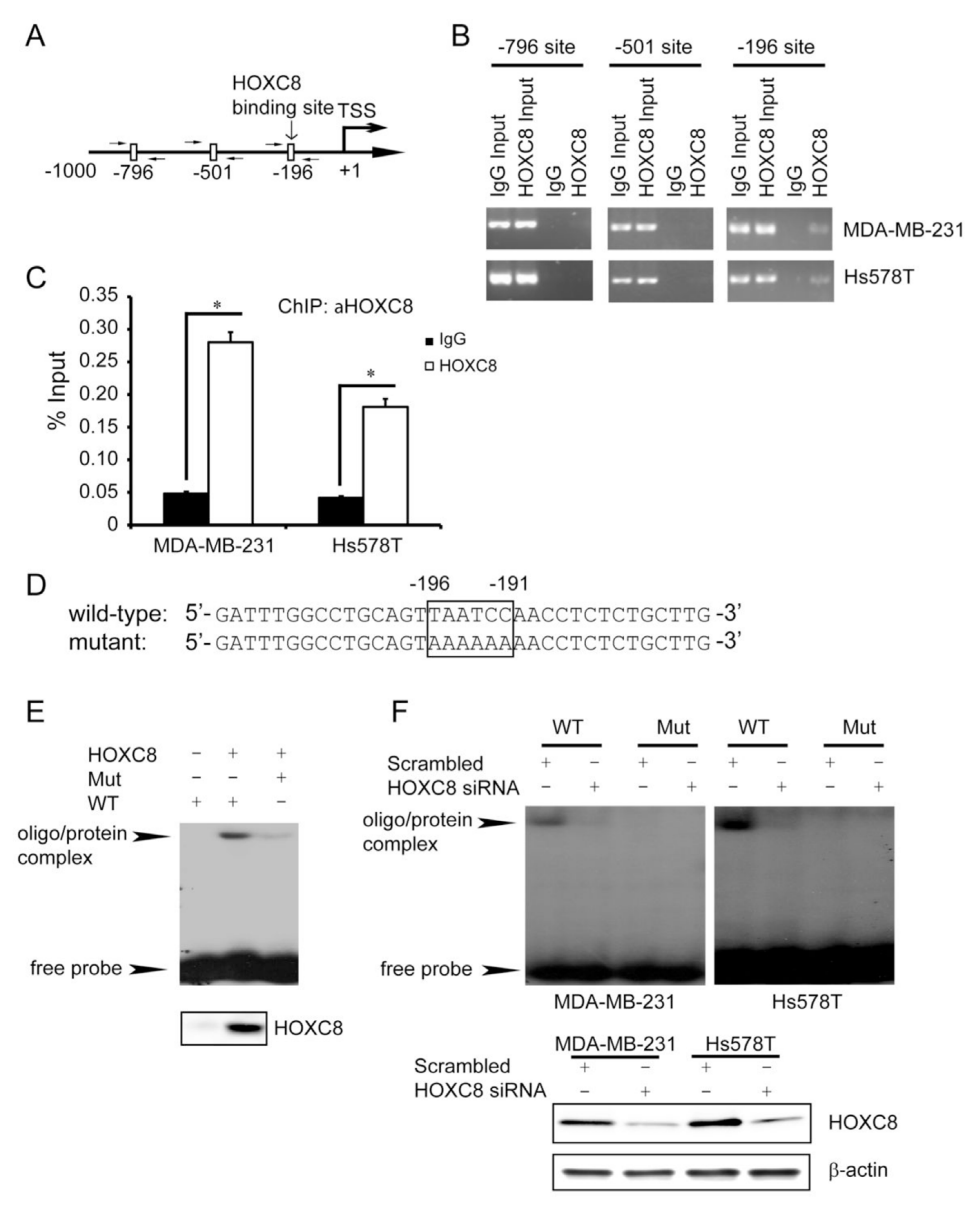
HOXC8 binds directly to CDH11 promoter *in vivo* and *in vitro* (A) Positions of PCR primers designed to amplify the regions containing putative HOX binding sites in the CDH11 promoter. (B) ChIP was performed on MDA-MB-231 or Hs578T cells using HOXC8 mAb or mouse IgG and the immunoprecipitated chromatin DNA was subjected to PCR using primers amplifying the putative HOX binding sites in the CDH11 promoter. (C) The HOXC8 mAb- or mouse IgG-immunoprecipitated chromatin DNA from MDA-MB-231 or Hs578T cells were subjected to qPCR using primer set amplifying the region containing nucleotides −196~−191. Columns, means; bars, SEM; *, *P* < 0.05. (D) The synthesized wild-type or mutant oligonucleotides that contain nucleotides −196~−191 of CDH11 promoter. (E) EMSA was performed using *in vitro*-translated HOXC8 protein and ^32^P-labeled wild-type or mutant oligonucleotides. The lower panel is the Western blots of HOXC8 protein *in vitro*-translated with TnT lysate system. (F) EMSA was performed using ^32^P-labeled wild-type or mutant oligonucleotides and nuclear extracts of control or HOXC8 knockdown cells. The lower panel is the Western blots of HOXC8 protein in control or HOXC8-knockdown cells.

To investigate *in vivo* HOXC8-CDH11 promoter interaction, we performed ChIP using HOXC8 mAb. Using primer sets that respectively amplify the regions containing nucleotides −196~−191, −501~−496 and −796~−791 of the CDH11 promoter (Fig.4A), PCR was only able to amplify the region containing nucleotides −196~−191, but not nucleotides −501~−496 or −796~−791 in the HOXC8 chromatin immunoprecipitates of MDA-MB-231 and Hs578T cells (Fig.4B). Quantitative PCR (qPCR) further showed that the region containing nucleotides −196~−191 was enriched approximately 6 and 4 fold in the HOXC8 chromatin immunoprecipitates of MDA-MB-231 and Hs578T cells respectively over their respective IgG control (Fig.4C). In a parallel experiment, we synthesized oligonucleotide corresponding to the nucleotides −211~−177 of CDH11 promoter and another oligonucleotide in which the TAATCC sequence was mutated to AAAAAA (Fig.4D). To determine whether HOXC8 was able to directly interact these oligonucleotides, we prepared *in vitro* translated HOXC8 protein using TnT lysate system. Electrophoretic mobility shift assay (EMSA) with ^32^P-labeled oligonucleotides showed the wild-type oligonucleotide was retarded by the HOXC8/TnT lysates while such retardation was absent in the empty vector/TnT lysates (Fig.4E). Moreover, mutant oligonucleotide was only minimally retarded by the HOXC8/TnT lysates (Fig.4E). In subsequent experiment, we carried out EMSA with nuclear extracts prepared from scrambled siRNA- or HOXC8 siRNA-treated MDA-MB-231 and Hs578T cells. ^32^P-labeled wild-type oligonucleode/protein complex was present in scrambled siRNA-treated cells but not in HOXC8 siRNA-treated cells (Fig.4F). In contrast, mutant oligonucleotide/protein complex was not detected in these cells (Fig.4F). Taken together, these results suggest that HOXC8 activates CDH11 transcription by directly binding to the TAATCC sequence at nucleotides −196~−191 of the CDH11 promoter.

### CDH11 mediates HOXC8-regulated anchorage-independent cell growth, migration, invasion and metastasis of breast cancer cells

**Figure5:**
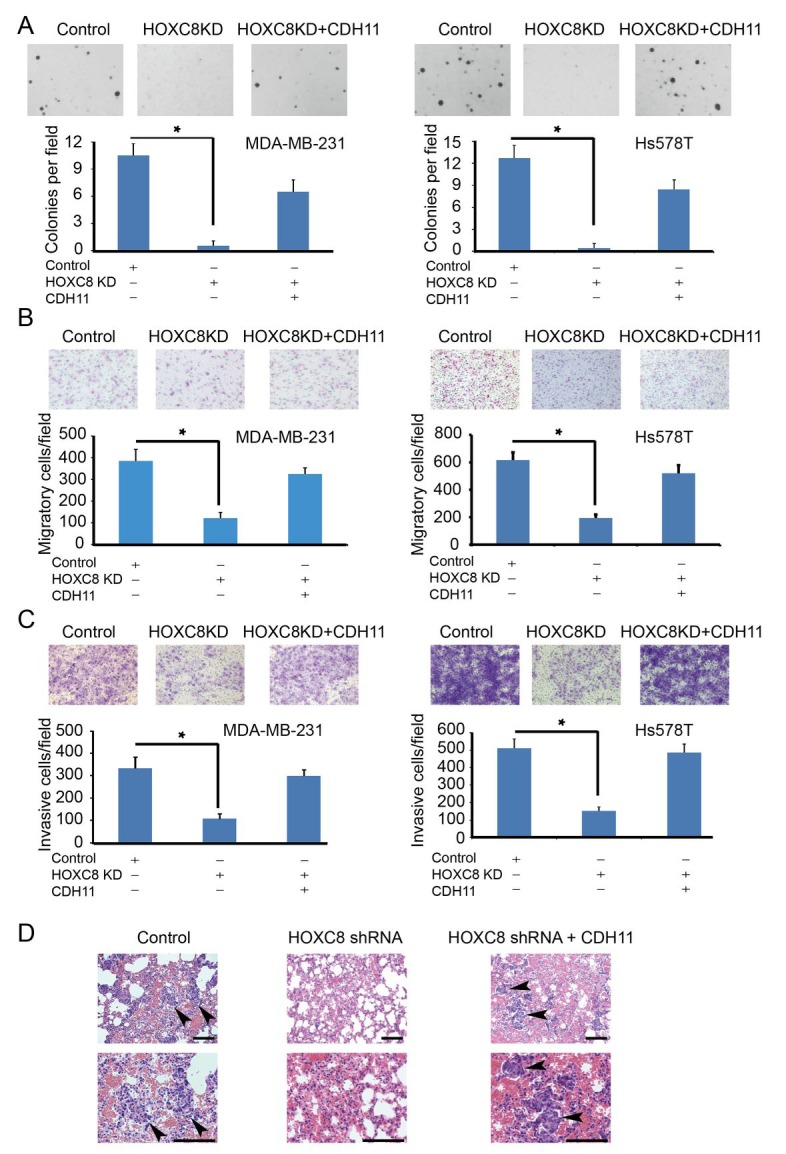
HOXC8-CDH11 pathway is critical for anchorage-independent cell growth, cell migration/invasion and spontaneous metastasis of breast cancer cells (A, B, C) Control (scrambled sequence), HOXC8-knockdown cells and HOXC8-knockdown cells with ectopic CDH11 expression were subjected to soft agarose colony formation (A), transwell cell migration (B), matrigel invasion assays (C). Columns, means; bars, SEM; *n* = 3; *, *P* < 0.05. (D) Control, HOXC8-knockdown MDA-MB-231 cells and HOXC8-knockdown MDA-MB-231 cells with ectopic CDH11 expression were injected into athymic female nude mice. After 7 weeks, mice lungs were fixed and sectioned. The sections were subjected to H&E staining and metastases were visualized under a light microscope. Arrows indicate metastases. Top: lower magnifications; bottom: higher magnifications; bar, 100 μm

Our previous work have demonstrated a critical role of HOXC8 in breast tumorigenesis because the depletion of HOXC8 resulted in the inhibition in cell migration, invasion and metastasis [7]. To investigate whether the tumorigenesis-promoting roles of HOXC8 is functionally linked to its ability to regulate CDH11 expression, we enforced CDH11 expression in HOXC8-knockdown MDA-MB-231 and Hs578T cells (Fig.S1). HOXC8-knockdown cells displayed impaired capability in anchorage-independent cell growth, cell migration and *in vitro* invasion compared to the control (Fig.5A, B and C). However, ectopically expressing CDH11 largely restored these events in HOXC8-knockdown cells (Fig.5A, B and C).

We next examined the importance of CDH11 in HOXC8-regulated tumorigenesis by subcutaneously injecting control, HOXC8-knockdown MDA-MB-231 cells and knockdown cells with ectopic CDH11 expression into the mammary fat pad area of female athymic nude mice. As we previously reported [7], silencing HOXC8 exhibited no obvious effect on *in vivo* tumor outgrowth (Fig.S2). Forced CDH11 expression in HOXC8-knockdown cells did little on the rate of tumor outgrowth (Fig.S2). At 7 weeks after tumor cell injection, mice were sacrificed and lungs were collected from these sacrificed animals. The collected lungs were sectioned and then subjected to H&E staining followed by the detection of metastatic lesions under microscope. Metastatic lesions were detected in the lungs of 70% of the mice receiving control cells (Table 1 and Fig.5D); in contrast, no metastatic lesion was seen in the lungs of mice receiving HOXC8-knockdown cells (Table 1 and Fig.5D). Ectopically expressing CDH11 largely restored the metastatic potential of the HOXC8-knockdown cells as metastatic lesion was observed in the lungs of 60% of the mice receiving HOXC8-knockdown cells with forced CDH11 expression (Table 1 and Fig.5D). These results thus support the notion that HOXC8 promotes breast metastasis through CDH11.

**Table 1:** Summary of Lung Metastases in Mice

	Number of mice with lung metastasis / total number of mice	metastasis lesions per slide
Control HOXC8 shRNA HOXC8 shRNA + CDH11	7/10 0/10 (P=0.0013)* 6/10 (P= 0.005)*	8± 3 0 (P < 0.0001)* 9± 4

* vs Control

### The expression of HOXC8 and CDH11 are associated and both expression correlates with poor recurrence-free survival of breast cancer patients

**Figure 6:**
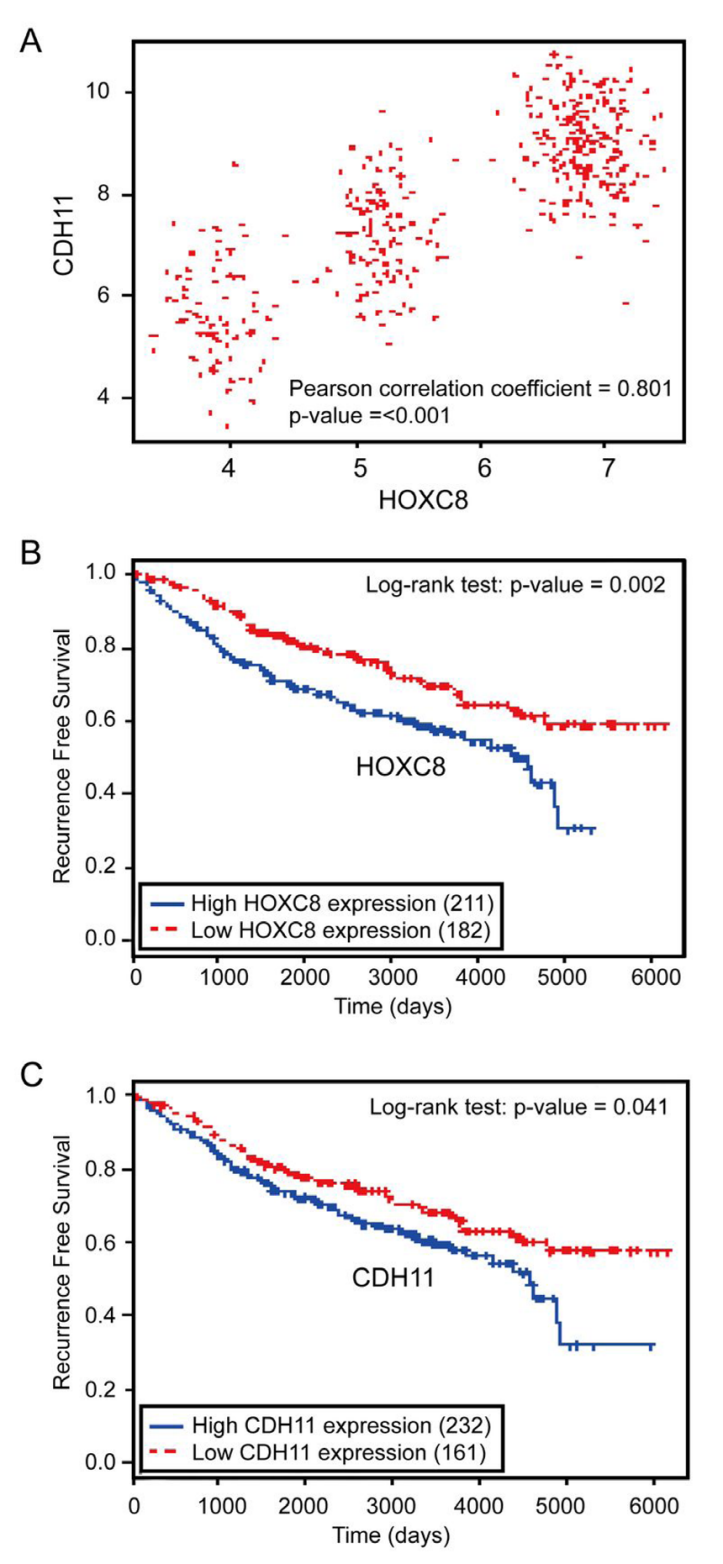
HOXC8 and CDH11 expressions are positively correlated and both are negatively associated with human breast cancer patient survival (A) The scatter plot of HOXC8 and CDH11 expression shows that they have in strong positive correlation (Pearson correlation coefficient = 0.801, *P* < 0.001). (B) Kaplan-Meier analysis of breast tumor gene expression database indicates a negative association between HOXC8 expression and recurrence-free survival of breast cancer patients (*P* = 0.002, log-rank test). (C) Kaplan-Meier analysis of breast tumor gene expression database indicates a negative association between CDH11 expression and recurrence-free survival of breast cancer patients (*P* = 0.041, log-rank test).

As laboratory study may not always recapitulate clinical breast malignancy, we explored the potential clinical relevance of HOXC8/CDH11 expression in human breast cancer. To do so, we first assessed the association between HOXC8 and CDH11 mRNA expression in breast tumors using microarray data of 414 breast cancer patients obtained from the Gene Expression Omnibus (GEO). Pearson correlation coefficient analysis showed that HOXC8 and CDH11 expressions were in a strong positive linear association (*ρ* = 0.801, *P* < 0.001; Fig.6A). Subsequently, we performed survival analysis (Kaplan-Meier method, log-rank test) to assess the potential correlation between HOXC8/CDH11 expression and recurrence-free survival of breast cancer patients. Both high HOXC8 and CDH11 expression were significantly associated with poor recurrence-free survival rate of breast cancer patients (*P* = 0.002 and 0.041 respectively) (Fig.6B and 6C). These results clearly support the experimental results that HOXC8 is a transcription factor of CDH11 and both are the positive regulators of breast tumorigenesis.

## DISCUSSION

A critical role of HOXC8 in tumor progression and development is implicated by the observation that the expression of HOXC8 is deregulated in various cancer types including prostate, cervical and breast cancer [5-7, 24]. Although the exact role of HOXC8 in tumorigenesis remains to be fully elucidated, the ability of HOXC8 to regulate the expression of genes essential for cell adhesion, migration and tumorigenesis [7, 25] indicates that it may impact tumor development by facilitating events toward tumor metastasis. This possibility is supported by our early finding that the depletion of HOXC8 diminishes breast cancer cell migration, invasion and metastasis [7].

The level of CDH11 is elevated in various cancers and its expression is closely associated with cancer cell migration and metastasis [5-6, 14, 17, 20]. For example, CDH11 is only expressed in invasive breast cancer cells while is absent in non-invasive ones [18, 22]. Forced expression of CDH11 has been shown to be sufficient to induce breast cancer cell migration and metastasis [19, 21]. Moreover, we previously reported that CDH11 was able to promote endogenous Rac activity by assisting the membrane localization of Rac-specific GEF Trio, an event important for Rac activation and breast cancer cell migration [22]. To understand the molecular mechanism mediating CDH11 expression in invasive breast cancer cells, we showed that knockdown of HOXC8 led to the reduction of CDH11 expression [22]. In this study, we further revealed that ectopically expressing HOXC8 alone is sufficient to enhance the level of CDH11 (Fig.1). Since modulating the abundance of HOXC8 affected the extent of RP-II occupancy in CDH11 promoter and CDH11 promoter activity (Fig.1 and 2), we reason that HOXC8 positively regulates CDH11 expression by enhancing CDH11 transcription.

All HOX proteins contain a highly conserved 60-amino-acid homeodomain that can bind DNA through TAATNN consensus sequence [1, 23]. HOXC8, as a member of HOX family proteins, has been previously shown to serve as a transcription to regulate the expression of osteopontin [26-27]. In this study, we showed that HOXC8 was able to directly bind to the TAATCC consensus sequence in the nucleotides −196~−191 of the CDH11 promoter (Fig.3). Moreover, we found that HOXC8 regulated CDH11 transcription through its binding to this sequence (Fig.3 and 4). Our study thus shows that CDH11 is a direct HOXC8 transcriptional target.

Our early study demonstrate that HOXC8 is required for breast cancer cell migration and metastasis [7]. Given the importance of CDH11 in breast cancer cell migration and metastasis [7, 22, 25], we speculated that HOXC8 might impact breast tumorigenesis by serving as a CDH11 transcription factor. Our reasoning is apparently supported by the observation that ectopically expressing CDH11 restored various tumorigenic events diminished in HOXC8-knockdown breast cancer cells (Fig.5 and Table 1). Strong correlation between HOXC8 and CDH11 expression in clinical breast tumor specimens (Fig.6) is also in agreement that HOXC8 is a CDH11 transcription factor. Moreover, the findings that both high HOXC8 and CDH11 expression correlate with poor recurrence-free survival of breast cancer patients further support the notion that the HOXC8-CDH11 functional axis plays a critical role in breast tumor progression and metastasis. In conclusion, our study indicates that novel and effective therapeutic approaches may be developed by targeting HOXC8-CDH11 functional axis.

## MATERIALS AND METHODS

### Cells and reagents

All breast cancer cell lines were obtained from American Type Culture Collection (ATCC, Manassas, VA) and cultured in DMEM supplemented with 10% fetal bovine serum (FBS). Anti-HOXC8 monoclonal antibody (mAb; titer, 1:1,000) and anti-HOXC8 rabbit polyclonal antibody (pAb; titer, 1:1000) were purchased from Abnova (Walnut, CA) and Sigma-Aldrich (St. Louis, MO) respectively. Anti-CDH11 mAb (titer, 1:1000) was obtained from Life Technology (Carlsbad, CA) and all other antibodies were obtained from Santa Cruz Biotechnology (Santa Cruz, CA). HOXC8 and CDH11 siRNA (ON-TARGET plus SMART pool) were purchased from ThermoFisher (Waltham, MA). TRIzol, Lipofectamin LTX and Lipofectamine 2000 were purchased from Life Technology. TnT coupled reticulocyte lysate systems were obtained from Promega (Madison, WI).

### CDH11 promoter reporters and luciferase assay

CDH11 promoter sequence was amplified using genomic DNA isolated from MDA-MB-231 cells (primer sequences are provided in Supplemental Table S1). Generated CDH11 promoter fragment was subcloned into the pGL3-basic vector (Promega) that contains the firefly luciferase reporter gene. The mutagenesis of HOXC8 binding site was generated through end-prolongation PCR as previously described (primer sequences are provided in Supplemental Table S1). To determine CDH11 promoter activity, overnight-cultured cells were transfected with CDH11 promoter reporter plasmids using lipofectamin LTX. PGK promoter-driven Renilla luciferase plasmid (Promega) was also included in the transfection to serve as an internal standard. Twenty-four hours after transfection, cells were washed with phosphate-buffered saline (PBS), lysed and luciferase activities were measured using the dual luciferase assay system (Promega). The CDH11 promoter activity is calculated by dividing the firefly luciferase activity with Renilla luciferase activity.

### *In vitro* transcription/translation of human HOXC8 protein

HOXC8 protein was synthesized in vitro using a TnT coupled transcription/translation reticulocyte lysate system according to manufacturer's protocol. To generate HOXC8 protein, human HOXC8 cDNA was subcloned into pcDNA3.1(+) and HOXC8 mRNA was transcribed using T7 RNA polymerase. The production of HOXC8 protein in TnT lysates was monitored by Western blot using anti-HOXC8 mAb.

### Electrophoretic mobility shift assay (EMSA)

EMSA was performed using nuclear extracts or aliquots from the TnT lysate reactions that contains the newly synthesized HOXC8 as previously described [28]. Briefly, ^32^P-labeled oligonucleotides were incubated with 10 μg of nuclear extracts or 1 μl of the TnT lysates at 37°C for 15 min followed by fractionating the reaction mixtures on a 6% polyacrylamide gel. Gel was dried and exposed to X-ray films.

### Chromatin Immunoprecipitation (ChIP)

ChIP assays were carried out as described previously [28]. Briefly, MDA-MB-231 or Hs578T cells were grown to near confluence in 15cm dishes and then fixed in 1% formaldehyde. Cells were scrapped from dishes, sonicated and precleared with protein A/G-agarose. Sheared chromatin was prepared, and immunoprecipitated with anti-HOXC8 or RNA polymerase II (RP-II) antibody overnight at 4°C. Immune complexes were captured using protein G-agarose, and the formaldehyde cross-links in the eluted complexes were reversed. The DNA was analyzed by PCR or real-time PCR. Sequences of the used primers are described in Supplemental Table S1.

### Construction CDH11 shRNA and CDH11 expression lentiviral vectors

HOXC8 shRNA sequences were generated with the aid of web-based Invitrogen Block-It program and expressed from pLV-shRNA vector (BioSettia, San Diego, CA). CDH11 lentiviral expression vector was developed by subcloning human CDH11 cDNA into pCDH-CMV-MCS-EF1-copGFP (System Biosciences, Mountain View, CA). Lentiviruses were prepared as previously described [29-30]. Sequences of HOXC8 are included in Supplemental Table S1.

### Soft agarose colony formation assay

The ability of MDA-MB-231 and Hs578T cells for anchorage-independent cell growth was determined by soft agarose colony formation assay as previously described [31]. Briefly, cells were first lentivirally transduced with HOXC8 shRNA to silence HOXC8 expression. The HOXC8-knockdown cells were then lenetivirally transduced with CDH11-expressing vector to enforce CDH11 expression. Generated cells were subjected to soft agarose colony formation assay in 6-well plates and 2 × 10^4^ cells were added into each well which consisted of a bottom base layer (0.6% agarose diluted in DMEM) and top layer (0.3% agarose diluted in DMEM). After 3 weeks, colonies were stained with iodonitrotetrazolium chloride (INT) and counted under a phase-contrast microscope.

### Transwell migration assays

Cell migration was performed as previously described [7, 22]. Briefly, the undersurface of upper chambers of Transwells (8 μm pore size; Costar) was coated with 10 μg/mL of Collagen I overnight at 4°C. Cells were detached with 0.05% Trypsin in PBS, suspended in serum-free medium at a density of 5 × 10^6^ cells/mL and 100 μL cell suspension was added into each upper chambers of Transwells. Meanwhile, 10% FBS was added into lower chamber to serve as chemo-attractants. After a 4-hour migration period, remaining cells in the upper chamber were removed with cotton swabs while cells on the undersurface of upper chambers were fixed with 5% glutaraldehyde and stained with crystal violet solution. The number of migratory cells was determined by counting the stained cells in three different fields under a phase-contrast microscope.

### *In vitro* invasion assay

Cell invasion was performed as previously described [7]. Cells were detached using 0.05% EDTA in PBS, suspended in serum-free medium and then plated into Matrigel-coated invasion chambers (Cell Biolabs, San Diego, CA) at 1.5 × 10^5^ cells/well. After 24 hours, the remaining cells in the chambers were removed by cotton swabs and the invading cells on the lower surface of the chambers were stained with Quick-Diff staining solution. The number of invading cells was determined by counting the stained cells in three different fields under a phase-contrast microscope.

### Mouse orthotopic breast tumor model

All animal studies were approved by the Georgia Regents University Institutional Animal Care and Use Committee. Mouse orthotopic breast tumor model was carried out as previously described [30]. Briefly, MDA-MB-231 cells (10^6^ cells in 0.1 mL PBS) were injected into the fourth mammary fat pad of athymic female nude mice at 4-6 weeks old (Harlan Laboratory, Tampa, FL) and animals were monitored for 7 weeks to determine *in vivo* tumor outgrowth. Tumor outgrowth was determined by measuring xenografts externally in two dimensions using a caliper and tumor volume (*V*) was calculated by equation: *V* = (*L* × *W*^2^) × 0.5, where *L* is the length and *W* is the width of a xenograft. To determine spontaneous tumor metastasis, mice were sacrificed at 7 weeks after injection. Lungs were removed, then fixed in 4% PBS-buffered paraformaldehyde and processed for paraffin-embedded sectioning. The sections were subjected to H&E staining and the metastatic lesions were visualized under a light microscope.

### Bioinformatics analysis

HOXC8 and CDH11 expressions in microarray data and survival data of breast cancer patients were obtained from the Gene Expression Omnibus (GEO) database (414 samples that are available for both HOXC8 and CDH11 expressions from GSE6532). To investigate the linear relationship between HOXC8 and CDH11 expressions, the Pearson correlation coefficient was employed. Kaplan-Meier method was used to estimate survival curves and the log-rank test was used to compare survival curves of high and low HOXC8 and CDH11 expression groups, respectively. Since the distributions of HOXC8 and CDH11 expressions were skewed in survival analysis,, all percentiles between the lower and upper quartiles of gene expression were computed for the log-rank test and the best performing threshold was used as a cut-off point for high and low groups of HOXC8 and CDH11 expressions respectively. For all statistical analyses, p < 0.05 were considered as significant.

### Statistical analysis

The data are presented as means ± SEM. Statistical analyses were performed on data collected from at least three independent experiments. Student's t-test (two-tailed) was used to compare two groups, and differences were considered statistically significant when *P* < 0.05.

## SUPPLEMENTARY FIGURES AND TABLE


