# Supplementary material for: HOXC8 promotes breast tumorigenesis by transcriptionally facilitating cadherin-11 expression
Source: Oncotarget. 2014 Mar 22;5(9):2596–607. doi: 10.18632/oncotarget.1841 (PMC4058030; doi:10.18632/oncotarget.1841)
Supplement: Supplementary file 1 [file oncotarget-05-2596-s001.pdf]

Table S1. Oligonucleotides used in real-time PCR, cloning and knockdown experiments

Primer Name	Sequence (5’ to 3’)
CDH11 promoter cloning	
-3000bp forward	GACGGCCTGCTTTGTGTTCAGGTGTCAGAC
-2000bp forward	TAAACCTGTCAGATCTCCTGAGACTTATTT
-1000bp forward	GACCTTGGGCTGGTTGGTTGGATATACTGT
-100bp forward	TCTGCGTGACGCGTCCGGGAGGCCACCCTC
Apa I reverse	AAGGGCCCTTTTGGTTACGTGGTAGGCACAGGAGAATG
ChIP primers	
CDH11 -796 forward	ATCTTCCTCCTTGAATTGAC
CDH11 -796 reverse	AGATCCCAAGAAACCATTGA
CDH11 -501 forward	CTAAACTGAACCCCAGTGTG
CDH11 -501 reverse	GCCTGGTACATAGTAAGCAC
CDH11 -196 forward	AGCCCGTAGCTCCCACGTGATA
CDH11 -196 reverse	CCAACTGTACGGTGGTCTTGCTGA
RP II forward	GCTAACTGGGGACGTGGGCA
RP II reverse	CAAAGGCATGGCTGTGGCAC
Knockdown shRNA	
Scrambled shRNA	AAAACAACAAGATGAAGAGCACCAATTGGATCCAATTGG TGCTCTTCATCTTGTTG
HOXC8 shRNA1	AAAAGCAATATCCCGACTGTAAATCTTGGATCCAAGATTT

HOXC8 shRNA2	ACAGTCGGGATATTGC<br><br>AAAAGCCTCATGTTTCCATGGATGATTGGATCCAATCATCC<br>TGGAAACATGAGGC
mutagenesis	
Forward 1<br><br>Reverse 1<br><br>Forward 2<br><br>Reverse 2	GACCTTGGGCTGGTTGGTTGGATATACTGT<br><br>TGGATTAACTGCAGGCCAAATCCCCAC<br><br>CAGTTAATCCAACCTCTCTCTGCTTGCAGAAG<br><br>AAGGGCCCTTTTGGTTACGTGGTAGGCACAGGAGAATG
Real-time PCR	
Cadherin 11 forward<br><br>Cadherin 11 reverse	GCATCCCGCCCATGAGTA<br><br>CGCACCCGCAGACTTTG

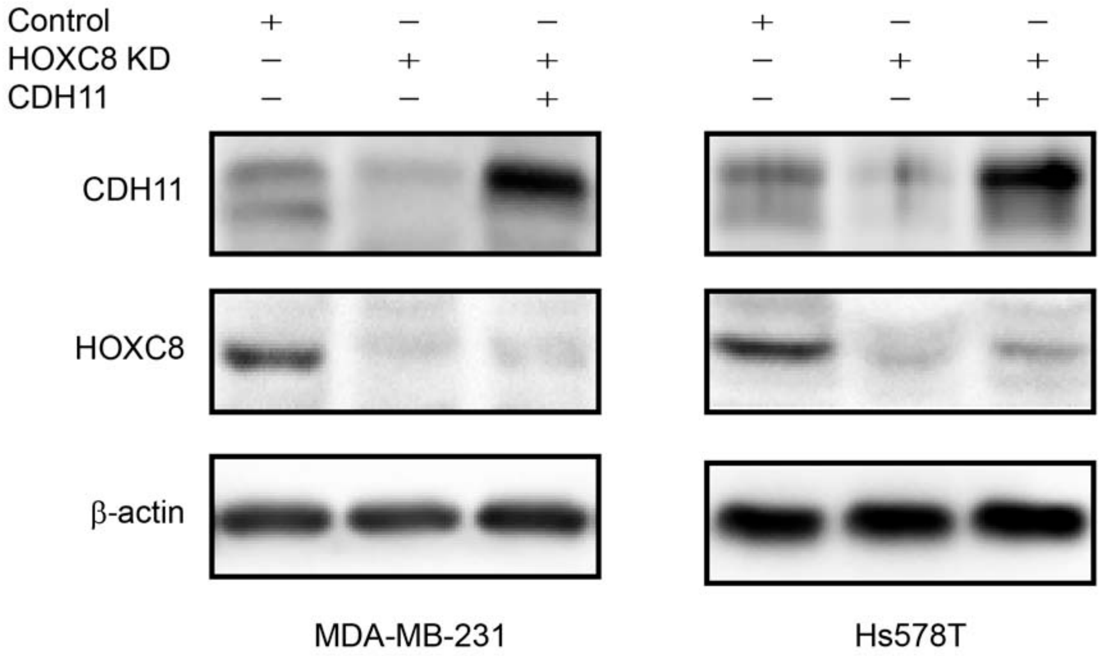

Figure S1. Expression of HOXC8 and CDH11 in control, HOXC8-knockdown cells and knockdown cells with ectopic CDH11 expression. Overnight culture cells were lysed for Western blot to detect HOXC8, CDH11 and β-actin with the respective antibodies.

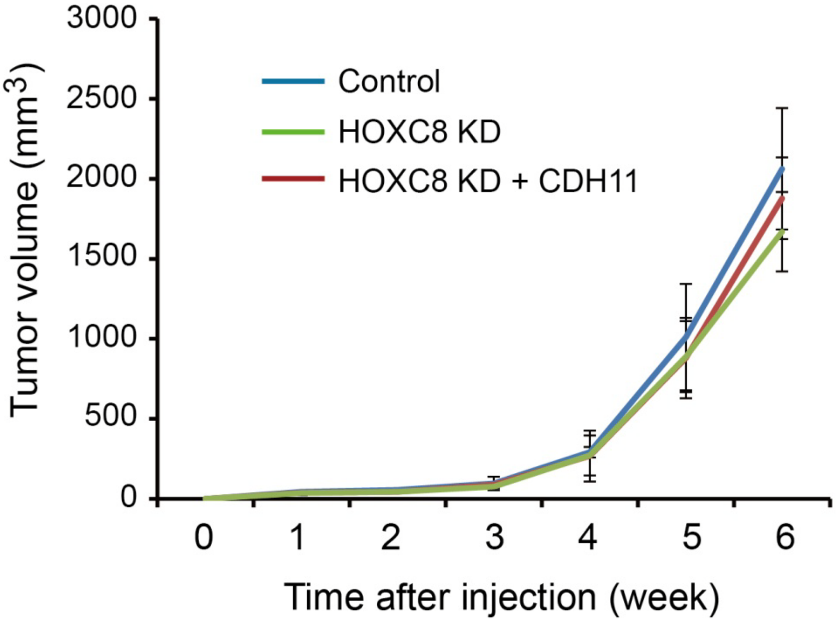

Figure S2. ***In vivo* tumor outgrowth of mice receiving control MDA-MB-231 cells, MDA-MB-231 cells lentivirally transduced with HOXC8 shRNA or HOXC8 shRNA plus CDH11 expression vector.** Cells (10^6 cells/mouse) were subcutaneously injected at 4th mammary fat pad area of female athymic nude mice (4-6 weeks of age). Xenografts were externally measured in two dimensions using a caliper. Data are means ± sem. n=10. Student-Newman-Keuls test were used to compare three groups. Conclusion: there was no statistically significant difference among control, HOXC8 KD and HOXC8 KD + CDH11 groups.
